# Effect of Tannin-Binding Agents (Polyethylene Glycol and Polyvinylpyrrolidone) Supplementation on *In Vitro* Gas Production Kinetics of Some Grape Yield Byproducts

**DOI:** 10.5402/2011/780540

**Published:** 2011-12-25

**Authors:** Maghsoud Besharati, Akbar Taghizadeh

**Affiliations:** Department of Animal Science, Faculty of Agriculture, University of Tabriz, Tabriz 51664, Iran

## Abstract

The effects of polyethylene glycol (PEG) and polyvinylpyrrolidone (PVP) on *in vitro* gas production characteristics, organic matter digestibility (OMD), and metabolizable energy (ME) contents of some grape yield byproducts were investigated. The gas production was recorded after 2, 4, 6, 8, 12, 16, 24, 36, and 48 h of incubation. The gas production profiles in triplicate fitted with equation Y = A (1 – e^−ct^). The data was analyzed using completely randomized design. Total phenol (TP) and total tannin (TT) contents were highest for raisin waste (RW). The TP content (g/kg DM) ranged from 30.1 in grape pomace (GP) to 96.3 in RW, which also had the higher TT (72.1 g/kg DM). The potential gas production (a + b) of DGB, GP, and RW were 239.43, 263.49, and 208.22 mL/g DM, respectively. In the absence of PEG and PVP, rate constant of gas production (c) for GP was highest among the feedstuffs (0.1073 mL/h), but in presence of PEG or PVP, RW had highest fraction (c) among the feedstuffs. Addition of PEG and PVP inactivated effects of tannins and increased gas production, ME, NE_1_, OMD, and VFA in grape yield byproducts. Addition of PEG and PVP could overcome adverse effects of tannins on nutrient availability as indicated by gas production parameters.

## 1. Introduction

A major constraint to increasing livestock productivity in developing countries is the scarcity and fluctuating quantity and quality of the year-round supply of conventional feeds. These countries experience serious shortages in animal feeds of the conventional type. In order to meet the projected high demand of livestock products and to fulfill the future hopes of feeding the millions and safeguarding their food security, the better utilization of nonconventional feed resources which do not compete with human food is imperative. There is also a need to identify and introduce new and lesser known food and feed crops. An important class of nonconventional feeds is byproduct feedstuffs which are obtained during harvesting or processing of a commodity in which human food or fibre is derived. The amount of byproduct feedstuffs generally increases as the human population increases and economies grow [1].

Several factors have lead to increase the interest in byproduct feedstuffs, such as pollution abatement and regulations, increasing costs of waste disposal, and changes in perception of the value of byproduct feedstuffs as economical feed alternatives [1].

The annually amount produced of agro-byproducts in Iran are generous, whereas, production of grape exceeds 2.87 million ton/yr, that proportion of grape yield is used for production of dried grape and grape juice. In this processes, dried grape byproduct (grape cluster stems plus rejected raisins) (DGB), raisin waste (RW), and grape pomace (GP) are produced in high level [2]. In developing countries, ruminants are fed low-quality roughages in various proportions depending on the type of animal and season. These feeds are poor in protein, energy, minerals, and vitamins. Addition of grape yield byproducts in ruminant diets can improve the utilization of low-quality roughages mainly through the supply of protein to rumen microbes, but the presence of tannins in these byproducts prevents not only their optimal utilization but also that of the roughages and byproducts. Addition of a tannin-complexing agent, polyvinylpyrrolidone (PVP), and polyethylene glycol (PEG) to tannin-rich diets is another attractive option to enhance the feeding value of such diets.

For about 3 decades, it has been known that tannins bind to PVP and PEG. PVP and PEG are also considered to break already formed tannin-protein complexes, as their affinity for tannins is higher than for proteins. This property of these tannin-complexing agents, in particular of PEG of molecular weight 3500 or 4000, has been exploited by various workers to alleviate the effects of tannins [3]. Addition of PEG results in the formation of PEG-tannin complexes which inactivates tannins. PVP and PEG of different molecular weights are available commercially. Systematic investigations were conducted on the binding efficiency of PVP (molecular weights: 10,000, 40,000, and 360,000) and PEG (molecular weight: 2000–35,000) in order to identify the most effective tannin-complexing agents [4]. The affinity of PVPs for tannins was lower than of PEGs. Furthermore, binding of insoluble PVP (PVPP) to tannins was lowest at pH = 7 and the binding with PEG 6000 was the same from pH = 4.7–7, except for quebracho tannins for which the binding increased as the pH approached 7. The binding with PEG 2000 decreased to a greater extent, as the pH reached near neutral, and for PEG 4000 this decrease was slightly lower. The PEGs were the most effective followed by PVPs and PVPP. The PEG 35,000 was the least effective amongst PEGs. The efficiency of other PEGs was similar. The PEG 6000 may be preferred for inactivation of tannins in feedstuffs as its binding to tannins was highest at near neutral pH values [3]. Addition of PEG to tannin-containing feeds increased *in vitro *gas and SCFA production and *in vitro *degradation of nitrogen. Therefore, there appears to be a potential for improving the utilization of tannin-containing feeds by the use of tannin-binding agent such as PEG without altering the genetic pool of tannin-containing plants. Inclusion of energy sources with the aim of synchronizing nitrogen degradability and availability of energy increased the efficiency of microbial protein synthesis in the presence of PEG [5]. This approach can be used both by farmers and by the industry. Farmers can give PEG directly to animals through water, by mixing it with a small amount of concentrate, by spraying it on tannin-rich feedstuffs or better still as a part of nutrient blocks. Industry can incorporate PEG in a pelleted diet composed of ingredients including tannin-rich byproduct(s) [4].

There is little information available on the nutritive value grape yield byproducts. Although grape pomace is low in ME, it has been used in diets of ruminants fed close to maintenance ME levels, especially sheep [6]. However, inclusion of grape pomace in the diet reduced digestibilities of the diet [7]. Lu and Yeap Foo [8] reported that grape pomace tannins have adverse effects on nutrient utilization, and are toxic at high intake levels [9] due to their ability to bind proteins, minerals, and carbohydrates [10]. Tannins are the most widely occurring antinutritional factor in nonconventional feeds.

The present study was carried out to study effect of adding PEG and PVP on *in vitro* gas production, metabolizable energy (ME), net energy for lactation (NE_l_), and organic matter digestibility (OMD) of grape pomace (GP), raisin waste (RW), and dried grape byproduct (DGB).

## 2. Materials and Methods

### 2.1. Grape Yield Byproducts

Grape yield byproducts were obtained from raisin and grape juice production factories of Tabriz, Iran. The DGB that was collected contained grape cluster stems and rejected raisins.

### 2.2. Chemical Composition

Feedstuffs dry matter (DM, method ID 934.01), ash (method ID 942.05), ether extract (EE, method ID 920.30), and crude protein (CP, method ID 984.13) were determined by procedures of AOAC [11]. The NDF and ADF concentrations were determined using the methods of Van Soest et al. [12] with sodium sulphite. NDF was analysed without amylase with ash included.

Total phenolics (TPs) were measured using the Folin-Ciocalteau method [13]. Total tannin (TT) was determined after adding insoluble polyvinylpyrrolidone and reacting with Folin-Ciocalteau reagent [13]. Tannic acid was used as the standard to express the amount of TP and TT.

### 2.3. *In Vitro* Gas Production Trial

The DM degradability of DGB, RW, and GP was determined by *in vitro* fermentation with ruminal fluid. Ruminal fluid was collected approximately 2 h after morning feeding from 2 cannulated sheep consuming 400 g alfalfa hay, 200 g barley, and 200 g soybean meal. Ruminal fluid was immediately squeezed through 4 layers of cheesecloth and was transported to the laboratory in a sealed thermos. The resulting ruminal fluid was purged with deoxygenated CO_2_ before use as the inoculum. Gas production was measured by Fedorak and Hurdy [14] method. Approximately, 300 mg of dried and ground (2 mm) DGB, RW, and GP samples with (300 mg) and without PEG (6000) or PVP (25000) were weighed and placed into serum bottles. Buffered rumen fluid with McDougall's buffer (20 mL) was pipetted into each serum bottle [15]. The gas production was recorded after 2, 4, 6, 8, 12, 16, 24, 36, and 48 h of incubation. Total gas values were corrected for the blank incubation, and reported gas values are expressed in mL per gram of DM. The gas production profiles in triplicate fitted with equation:

(1) $$Y=A\left(1-e^{-\text{ct}}\right),$$

where *Y* is the volume of gas production (mL/g DM) at time *t*, *A* is gas production from soluble and insoluble fraction, c is the gas production rate, and *t* is the incubation time (h). The ME contents of GP and OMD were calculated using equations of Menke et al. [16] as:

(2) $$\begin{array}{cc} & \text{ME},\text{MJ}/\text{kg}\;\text{DM}\\ & \;\;=2.20+0.136\times \text{Gv}+0.057\times \text{CP}+0.0029\times {\text{CP}}^{2},\\ & \text{OMD},\text{g}/100\;\text{g}\;\text{DM}\\ & \;\;=14.88+0.889\times \text{Gv}+0.45\times \text{CP}+0.0651\times \text{XA}, \end{array}$$

where OMD = OM digestibility (g/100 g DM), XA = ash in g/100 g DM, and Gv = the net gas production (mL) at 24 h. The VFA were calculated using the equation below as:

(3) VFA,mmol=−0.00425+0.0222 Gv,

and NE_l_ was calculated using equation as:

(4) $$\begin{array}{cc} & {\text{NE}}_{\text{l}}\;\left(\text{Mcal}/\text{lb}\right)\\ & \;=\frac{\left(2.20+\left(0.0272\times \text{Gas}\right)+\left(0.057\times \text{CP}\right)+\left(0.149\times \text{CF}\right)\right)}{14.64}, \end{array}$$

where Gas is 24 h net gas production (mL/g DM), CP is crude protein (% of DM), and CF is crude fat (% of DM).

### 2.4. Statistical Analysis

Data obtained from this study was subjected to ANOVA as a completely randomized design with 3 replicates by the GLM procedure [17], and treatment means were compared by the Duncan test.

## 3. Results

The chemical composition of feeds is shown in Table 1. All of the grape yields byproducts in this experiment had the same CP content, approximately. Grape pomace had the lowest ADF and NDF contents within the grape yield byproducts. Total phenols and total tannin contents were highest for raisin waste. The TP content (g/kg DM) ranged from 30.1 in grape pomace to 96.3 in raisin waste (Table 1), which also had the higher TT (72.1 g/kg DM).

Total gas production volume of feedstuffs in incubation times (mL/g DM) are presented in Table 2. Addition of PEG or PVP to tannin-containing feeds increased *in vitro *gas production in all feeds. At the 2 h incubation times, the gas production volume of GP, GP + PEG, and GP + PVP were 39.2, 7.6, and 9.4 mL/g DM, for RW, RW + PEG, and RW + PVP were 33.1, 12.7, and 9.8 mL/g DM, for DGB, DGB + PEG, and DGB + PVP were 32.6, 6.8, and 6.9 mL/g DM, respectively. At the first incubation times (2 and 4 h), the control treatments (treatment without PEG or PVP) had the highest *in vitro* gas production volume within treatment (*P* < 0.05). At the 6 h of incubation times, except GP, for the other byproducts gas production volume were approximately the same within treatments. After 6 h incubation time, the treatments with PEG or PVP had the highest gas production in compared with control treatment (treatment without PEG or PVP; *P* < 0.05).

**Figure 1:**
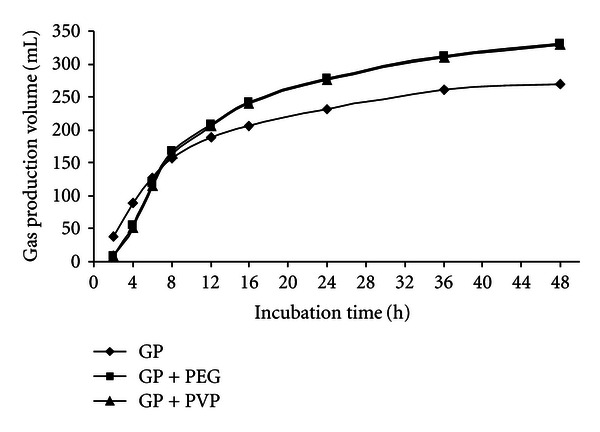
The effect of PEG and PVP on gas production of grape pomace.

**Figure 2:**
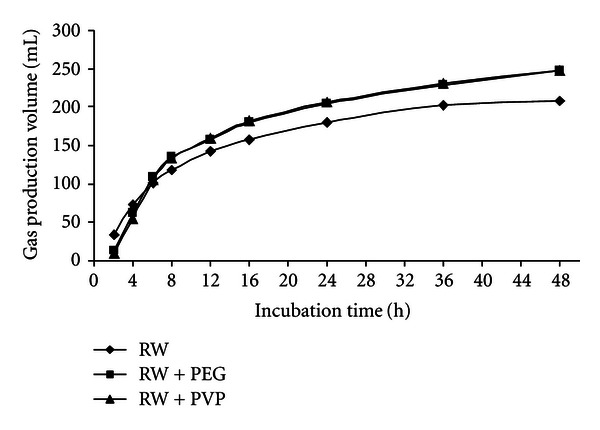
The effect of PEG and PVP on gas production of raisin waste.

**Figure 3:**
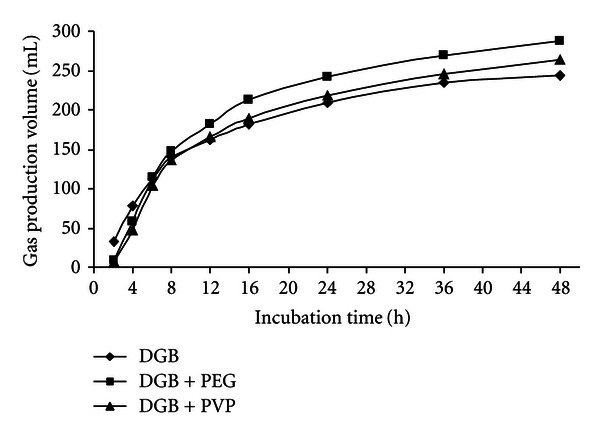
The effect of PEG and PVP on gas production of dried grape byproduct.

At the 48 h incubation times, the gas production volume of GP, GP + PEG, and GP + PVP were 269.8, 311.1, and 327.8 mL/g DM, respectively, for RW, RW + PEG, and RW + PVP were 208.7, 247.1, and 247.9 mL/g DM, respectively, for DGB, DGB + PEG, and DGB + PVP were 243.4, 286.5 and 264.1 mL/g DM, respectively. Figures 1, 2, and 3 show the pattern of *in vitro* gas production of the feedstuffs.

**Figure 4:**
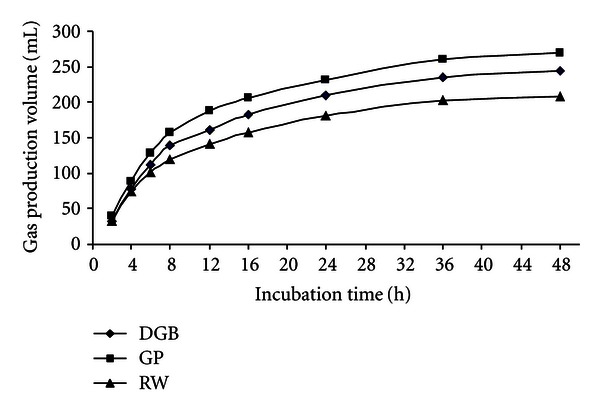
The gas production volume of grape yield byproducts.

**Figure 5:**
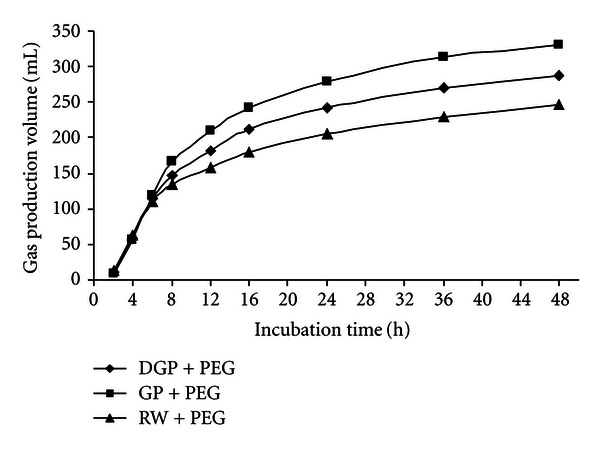
The gas production volume of grape yield byproducts with polyethylene glycol.

**Figure 6:**
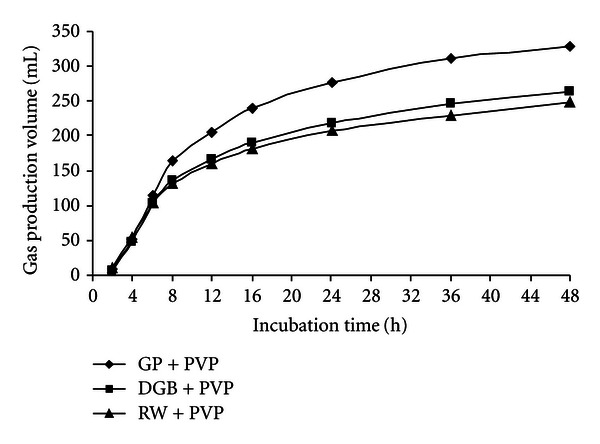
The gas production volume of grape yield byproducts with polyvinylpyrrolidone.

Statistical comparisons within grape yield byproducts for gas production volume (mL/g DM) are shown in Table 3. In control statement (without adding PEG or PVP), GP had the highest gas production volume at the all incubation times within the grape yield byproducts (*P* < 0.05) and at the 48 incubation time gas production of GP was 269.8 mL/g DM. Within the grape yield byproducts, gas production volume of RW was the lowest (*P* < 0.05). The ranking of feedstuffs on the basis of gas production was as follows: grape pomace > dried grape byproduct > raisin waste. When the PEG or PVP were added to byproducts, gas production improved but the gas production of GP was highest yet (*P* < 0.05). Figures 4, 5, and 6 show comparisons of gas production volume within grape yield byproducts.

The parameters estimated from the gas production of grape yield byproducts, with or without PEG or PVP, are given in Table 4. The PEG and PVP supplementation had also a significant effect on the estimated parameters of OMD, ME, NE_l_, and VFA (Table 4). In all byproducts except of RW, PEG, and PVP improved the amounts of OMD, ME, NE_l_, and VFA. For RW difference within control and treatment with PVP was not significant (*P* > 0.05). In all byproducts, PEG and PVP increased the amounts of potential gas production (a + b) and in all byproducts except of RW, PEG, and PVP decreased the amount of rate constant of gas production during incubation (c). Estimated variable in byproducts was higher for samples incubated in presence of PEG as compared to PVP. Addition of PEG significantly (*P* < 0.05) increased production of total VFA (from 13.02% in RW and to 21.02% in GP).

Statistical comparison within the parameters estimated from the gas production in grape yield byproducts are shown in Table 5. The potential gas production (a + b) of DGB, GP, and RW were 239.43, 263.49, and 208.22 mL/g DM, respectively. In the absence of PEG and PVP, rate constant of gas production (c) for GP was highest among the feedstuffs (0.1073 mL/h), but in presence of PEG or PVP, RW had highest fraction (c) among the feedstuffs. Estimated MEs from gas production for DGB, RW, and GP were 11.63, 10.79, and 12.4 MJ/kg DM that GP had biggest ME content among byproducts and ME content for RW was smallest (*P* < 0.05). For NE_l_ difference between DGB and RW was not significant (*P* > 0.05). The VFA ranged from 0.799 to 1.024 mmol.

## 4. Discussion

Total phenols and total tannin contents in GP were 30.1 and 22.7 g/kg DM, respectively, which are greater than the amounts that reported by Alipour and Rouzbehan [18] for grape pomace (22.7 and 15.6 g/kg DM, resp.).

The PEG or PVP supplementation had significant effect on *in vitro* gas production of DGB, GP, and RW (Table 2). These results are in agreement with the findings of Getachew et al. [19], Getachew et al. [20], Seresinhe and Iben [21], and Singh et al. [22]. Tannins bind to protein and decrease accessibility of proteins to rumen microorganisms. Tannins may form a less digestible complex with dietary proteins and may bind and inhibit the endogenous protein, such as digestive enzymes [23]. Tannin can adversely affect the microbial and enzyme activities [24–27]. Hagerman et al. [28] reported that tannins reduced CP digestibility. In another study, McNeill et al. [29] showed that by increasing condensed tannin in diet (from 6 to 65 g/kg DM), N digestibility decreased from 0.805 to 0.378 and excretory N in sheep feces increased from 4.3 to 9.7 g/d. Besharati and Taghizadeh [2] showed that addition of DGB to basal diets had effect on digestibility of CP (*P* < 0.05), also increasing of DGB supplementation level had linear effect on CP digestibility of diets (*P* < 0.05). The substantial reduction in N digestibility as a result of the presence of tannins was similar to that reported in sheep fed Lotus pedunculatus as a sole diet [30] and when Lotus pedunculatus was fed with ryegrass (*Lolium perenne*) [31], with and without polyethylene glycol (PEG). PEG, a nonnutritive synthetic polymer, has a high affinity to tannins and makes tannins inert by forming tannin PEG complexes [4]. PEG can also liberate protein from the preformed tannin-protein complexes [32]. The increase in the gas production in the presence of PEG is possibly due to an increase in the available nutrients to rumen micro-organisms, especially the available nitrogen. McSweeney et al. [33] showed that the addition of PEG caused a significant and marked increase in the rate and extent of ammonia production in the rumen. Tannins also have effects on carbohydrates, particularly hemicellulose, cellulose, starch, and pectins [34]. PEG and PVP supplementation increased the potential gas production (a + b), whereas PEG and PVP supplementation decreased the gas production rate in GP and DGB (*P* < 0.05). This result could suggest that tannins in this case are binding to fibres, and the presence of PEG increased microbial plant adhesion and/or the fibrolytic microbial activity. However, the PEG and PVP supplementation induce a decrease in rate of gas production in DGB and GP. This result has also been reported by Frutos et al. [35] and Guimarãez-Beelen et al. [36]. The latter authors have noted that for species, which the rate of gas production is reduced, the bacteria colonization is restricted. This could suggest that complexes forming between tannins and PEG generate steric obstruction which do not permit and/or limit the fixation of adherent bacteria to the feeds. Canbolat et al. [37] reported that PEG supplementation increased the gas production from the insoluble fraction (b), whereas PEG supplementation had no effect on the gas production from the immediately soluble fraction (a), and the gas production rate (c).

On the other hand, there were significant increases in the OMD and ME content of grape yield byproducts. These results are in agreement with the findings of Getachew et al. [19], Getachew et al. [20] and Seresinhe and Iben [21]. Rubanza et al. [38] reported that the increase in ME of leaves from Acacia species due to PEG (100 mg) ranged from 2.0 to 7.1 ME units. Similarly, Rubanza et al. [39] reported that PEG inclusion increased the ME values of leaves from browse fodders from 0.33 to 1.56 ME units. Adding PEG and PVP improved VFA content in all samples (Table 4). McSweeney et al. [33] showed that addition of PEG caused a significant and marked increase in the rate and extent of ammonia production. Stienezen et al. [40] showed that PEG caused a substantial increase in rumen ammonia concentration relative to the sheep receiving tannin (258 versus 155 *μ*mol/mL) and increased N digestibility from 0.631 to 0.776 (*P* < 0.001). They showed, fecal N concentrations were much greater (*P* < 0.001) in sheep receiving tannin than those receiving polyethylene glycol (PEG). Priolo et al. [41] reported the greater ruminal ammonia and a VFA concentration in PEG- versus tannin-fed sheep indicates more rapid ruminal fermentation when PEG was given.

Addition of PEG to tannin-containing feeds increased *in vitro *gas and SCFA production, and *in vitro *degradation of nitrogen. Therefore, there appears to be a potential for improving the utilization of tannin-containing feeds by the use of tannin-binding agent such as PEG without altering the genetic pool of tannin-containing plants. Inclusion of energy sources with the aim of synchronizing nitrogen degradability and availability of energy increased the efficiency of microbial protein synthesis in the presence of PEG [5].

## 5. Conclusion

Addition of PEG and PVP could overcome adverse effects of tannins on nutrient availability as indicated by gas production parameters. Addition of PEG and PVP inactivated effects of tannins and increased gas production, ME, NE_l_, OMD, and VFA in some tannin-containing feedstuffs. However there is a lack of information about feasibility of using PEG and PVP in tannin-rich diets for ruminants. PEG and PVP supplementation to improve the nutritive value of grape yield byproducts should be further analyzed in detail whether or not it is economical due to high price of PEG and PVP, before large scale implementation. However, Makkar [3] reported that some other substances such as wood ash, NaOH, and urea can be used instead of PEG.

## Figures and Tables

**Table 1:** The chemical composition of feeds (g/kg DM)^a^.

Feeds	DM	CP	NDF	ADF	Crude fat	OM	Total phenols	Total tannins
Grape pomace	933	66.2	187	184	14.1	877	30.1	22.7
Raisin waste	916	62.4	280	276	12.3	927.7	96.3	72.1
Dried grape byproduct	884.5	63.5	259	255	11.2	926	67	52.3

^a^DM: dry matter, CP: crude protein, NDF: neutral detergent fiber, ADF: acid detergent fiber, and OM: organic matter.

**Table 2:** Total gas production volume of grape yield byproducts in incubation times (mL/g DM).

Treatments	Incubation times (h)								
	2	4	6	8	12	16	24	36	48
Grape pomace (GP)									
Control	39.2^a^	89.1^a^	127.8^a^	157.7^b^	188.8^b^	206.3^b^	231.7^b^	260.3^b^	269.8^b^
GP + PEG	7.6^b^	56.2^b^	119.1^b^	166.7^a^	208.6^a^	242.0^a^	277.8^a^	312.5^a^	331.1^a^
GP + PVP	9.4^b^	51.3^b^	115.5^b^	163.6^a^	205.4^a^	239.8^a^	276.5^a^	310.5^a^	327.8^a^
SEM	1.59	1.59	1.49	1.25	1.83	2.36	2.45	2.39	2.54
Raisin waste (RW)									
Control	33.1^a^	73.8^a^	101.0^b^	118.7^b^	141.8^b^	156.6^b^	180.9^b^	203.2^b^	208.7^b^
RW + PEG	12.7^b^	62.0^b^	109.6^a^	134.3^a^	158.4^a^	180.3^a^	204.4^a^	229.4^a^	247.1^a^
RW + PVP	9.8^b^	54.4^c^	104.6^ab^	132.8^a^	159.9^a^	182.3^a^	206.5^a^	229.7^a^	247.9^a^
SEM	1.41	1.49	1.85	2.51	3.51	3.74	3.87	3.64	3.54
Dried grape byproduct (DGB)									
Control	32.7^a^	77.5^a^	112.7^a^	139.1^a^	161.6^b^	182.5^b^	209.2^b^	234.5^b^	243.4^b^
DGB + PEG	9.8^b^	58.4^b^	114.8^a^	146.9^a^	182.5^a^	212.3^a^	241.8^a^	269.2^a^	286.5^a^
DGB + PVP	6.9^b^	47.8^c^	103.3^b^	136.3^a^	165.6^b^	189.9^b^	218.3^b^	234.5^b^	264.1^b^
SEM	2.01	2.83	2.50	3.59	4.02	5.16	6.08	6.12	6.18

GP: grape pomace, RW: raisin waste, and DGB: dried grape pomace.

^a,b,c^Within a column, means without a common superscript letter differ (*P* < 0.05).

**Table 3:** Statistical comparison in grape yield byproducts for gas production volume (mL/g DM).

Treatments	Incubation times (h)								
	2	4	6	8	12	16	24	36	48
Control									
DGB	32.7^b^	77.5^b^	112.7^b^	139.1^b^	161.6^b^	182.5^b^	209.2^b^	234.5^b^	243.4^b^
GP	39.2^a^	89.1^a^	127.8^a^	157.7^a^	188.8^a^	206.3^a^	231.7^a^	260.3^a^	269.8^a^
RW	33.1^b^	73.8^c^	101.0^c^	118.7^c^	141.8^c^	156.6^c^	180.9^c^	203.2^c^	208.7^c^
SEM	0.71	0.78	0.979	1.366	1.76	1.749	2.425	3.187	3.613
Byproducts + PEG									
DGB	9.8^a^	58.4^a^	114.8^a^	146.9^b^	182.5^b^	212.3^b^	241.8^b^	269.2^b^	286.5^b^
GP	7.6^a^	56.2^a^	119.1^a^	166.7^a^	208.6^a^	242.0^a^	277.8^a^	312.5^a^	331.1^a^
RW	12.7^a^	62.0^a^	109.6^a^	134.3^c^	158.4^c^	180.3^c^	204.4^c^	229.4^c^	247.1^c^
SEM	1.97	3.09	2.739	3.072	4.132	4.651	5.198	4.599	4.293
Byproducts + PVP									
DGB	6.9^a^	47.8^b^	103.3^b^	136.3^b^	165.6^b^	189.9^b^	218.3^b^	234.5^b^	264.1^b^
GP	9.4^a^	51.3^ab^	115.5^a^	163.6^a^	205.4^a^	239.8^a^	276.5^a^	310.5^a^	327.8^a^
RW	9.8^a^	54.4^a^	104.6^b^	132.8^b^	159.9^b^	182.3^b^	206.5^b^	229.7^b^	247.9^b^
SEM	2.06	1.60	1.857	3.081	3.427	4.636	5.015	5.017	5.064

GP: grape pomace, RW: raisin waste, and DGB: dried grape pomace.

^a,b,c^Within a column, means without a common superscript letter differ (*P* < 0.05).

**Table 4:** The parameters estimated from the gas production of grape yield byproducts with or without PEG or PVP.

Treatments	Estimated parameters					
	a + b	c	ME	OMD	NE_l_	VFA
Grape pomace (GP)						
Control	263.49^c^	0.1073^a^	12.40^b^	59.85^b^	0.406^b^	1.024^b^
GP + PEG	317.03^a^	0.0921^c^	13.66^a^	68.05^a^	0.423^a^	1.229^a^
GP + PVP	314.11^b^	0.0972^b^	13.62^a^	67.82^a^	0.422^a^	1.223^a^
SEM	0.118	0.0003	0.067	0.436	0.001	0.011
Raisin waste (RW)						
Control	208.22^b^	0.0976^c^	10.79^b^	50.32^b^	0.367^b^	0.799^b^
RW + PEG	235.86^a^	0.1082^a^	11.43^a^	54.50^a^	0.376^a^	0.903^a^
RW + PVP	235.66^a^	0.1028^b^	11.49^a^	54.89^a^	0.376^a^	0.913^a^
SEM	0.0955	0.00014	0.105	0.688	0.001	0.017
Dried grape byproduct (DGB)						
Control	239.43^c^	0.0992^a^	11.63^b^	55.41^b^	0.367^b^	0.924^b^
DGB + PEG	280.59^a^	0.0939^c^	12.51^a^	61.22^a^	0.379^a^	1.069^a^
DGB + PVP	252.24^b^	0.0966^b^	11.87^b^	57.03^b^	0.370^b^	0.965^b^
SEM	0.277	0.00003	0.166	1.082	0.002	0.027

GP: grape pomace, RW: raisin waste, DGB: dried grape pomace, ME: metabolizable energy (MJ/kg DM), OMD: organic matter digestibility (g/100 g DM), NE_l_: net energy for lactation (Mcal/lb), VFA: volatile fatty acids (mmol), (a + b): potential gas production (mL/g DM) and c: rate constant of gas production during incubation (mL/h).

^a,b,c^Within a column, means without a common superscript letter differ (*P* < 0.05).

**Table 5:** Statistical comparison within the parameters estimated from the gas production in grape yield byproducts.

Treatments	Estimated parameters					
	a + b	c	ME	OMD	NE_l_	VFA
Control						
DGB	239.43^b^	0.0992^b^	11.63^b^	55.41^b^	0.367^b^	0.924^b^
GP	263.49^a^	0.1073^a^	12.40^a^	59.85^a^	0.406^a^	1.024^a^
RW	208.22^c^	0.0976^c^	10.79^c^	50.32^c^	0.367^b^	0.799^c^
SEM	0.0299	0.00017	0.066	0.431	0.001	0.011
Byproducts + PEG						
DGB	280.59^b^	0.0939^b^	12.51^b^	61.22^b^	0.379^b^	1.069^b^
GP	317.03^a^	0.0921^c^	13.66^a^	68.05^a^	0.423^a^	1.229^a^
RW	235.86^c^	0.1081^a^	11.43^c^	54.50^c^	0.376^b^	0.903^c^
SEM	0.0751	0.00013	0.141	0.924	0.002	0.023
Byproducts + PVP						
DGB	252.24^b^	0.0966^b^	11.87^b^	57.03^b^	0.370^b^	0.965^b^
GP	314.12^a^	0.0972^b^	13.62^a^	67.82^a^	0.422^a^	1.223^a^
RW	235.66^c^	0.1028^a^	11.49^b^	54.89^b^	0.376^b^	0.913^b^
SEM	0.14304	0.00022	0.136	0.981	0.002	0.022

GP: grape pomace, RW: raisin waste, DGB: dried grape pomace, ME: metabolizable energy (MJ/kg DM), OMD: organic matter digestibility (g/100 g DM), NE_l_: net energy for lactation (Mcal/lb), VFA: volatile fatty acids (mmol), (a + b): potential gas production (mL/g DM) and c: rate constant of gas production during incubation (mL/h).

^a,b,c^Within a column, means without a common superscript letter differ (*P* < 0.05).
